# Biochemical Properties of a Decoy Oligodeoxynucleotide Inhibitor of STAT3 Transcription Factor

**DOI:** 10.3390/ijms19061608

**Published:** 2018-05-30

**Authors:** David S. Lee, Rachel A. O’Keefe, Patrick K. Ha, Jennifer R. Grandis, Daniel E. Johnson

**Affiliations:** 1 School of Medicine, University of California at San Francisco, San Francisco, CA 94115, USA; dlee@ucsf.edu; 2 Department of Otolaryngology—Head and Neck Surgery, University of California at San Francisco, San Francisco, CA 94115, USA; rachel.okeefe@ucsf.edu (R.A.O.); patrick.ha@ucsf.edu (P.K.H.); jennifer.grandis@ucsf.edu (J.R.G.)

**Keywords:** STAT3 as a drug target, cyclic STAT3 decoy, oligodeoxynucleotide inhibitor, head and neck cancer

## Abstract

Cyclic STAT3 decoy (CS3D) is a second-generation, double-stranded oligodeoxynucleotide (ODN) that mimics a genomic response element for signal transducer and activator of transcription 3 (STAT3), an oncogenic transcription factor. CS3D competitively inhibits STAT3 binding to target gene promoters, resulting in decreased expression of proteins that promote cellular proliferation and survival. Previous studies have demonstrated antitumor activity of CS3D in preclinical models of solid tumors. However, prior to entering human clinical trials, the efficiency of generating the CS3D molecule and its stability in biological fluids should be determined. CS3D is synthesized as a single-stranded ODN and must have its free ends ligated to generate the final cyclic form. In this study, we report a ligation efficiency of nearly 95 percent. The ligated CS3D demonstrated a half-life of 7.9 h in human serum, indicating adequate stability for intravenous delivery. These results provide requisite biochemical characterization of CS3D that will inform upcoming clinical trials.

## 1. Introduction

Signal transducer and activator of transcription 3 (STAT3) is a highly regulated transcription factor that plays a prominent role in cellular growth and survival, as well as inflammation [1,2]. STAT3 is activated by phosphorylation of tyrosine 705 by upstream kinases, including Janus kinases (JAKs). Phosphorylation of STAT3 leads to homodimerization, translocation to the nucleus, and induction of target gene expression. In normal cells, activation of STAT3 is typically transient. However, constitutive hyperactivation of STAT3 has been observed in multiple human cancers, where it contributes to tumor development [3,4]. Additionally, STAT3 signaling promotes resistance to conventional chemoradiation and small-molecule inhibitors of other oncogenic signaling pathways [5,6,7,8,9,10,11]. Not surprisingly, aberrant activation of STAT3 has been shown to correlate with poor clinical prognosis in a number of malignancies [12,13,14]. Preclinical studies indicate that knockdown or inhibition of STAT3 selectively causes apoptosis in cancerous cells and rescues sensitivity to the aforementioned therapies [3,4,15,16,17]. By contrast, abrogation of STAT3 signaling is substantially less deleterious to noncancerous cells, at least in healthy adults [2,18]. In view of the pivotal role of STAT3 in human malignancies, a concerted effort has been made over the past two decades to develop selective inhibitors of STAT3 signaling.

The blockade of upstream kinases (e.g., JAKs) is a well-validated strategy for inhibiting STAT3 activation and inducing anticancer effects [9,19,20,21,22,23]. Indeed, most currently advertised STAT3 inhibitors target upstream activators. More recently, pharmaceuticals have been developed that interfere with expression of the STAT3 protein [22,24]. Among these is AZD9150, a modified antisense oligodeoxynucleotide (ODN) that targets *STAT3* mRNA for destruction. AZD9150 has shown promise in early clinical trials, reducing tumor burden in patients with refractory lymphoma and non-small cell lung cancer [24]. It is currently being evaluated as monotherapy in patients with advanced solid tumors and in combination with chemotherapy and/or durvalumab, an anti-PD-L1 monoclonal antibody (NCT: 03421353).

STAT3 decoys utilize a distinct mechanism of action to inhibit signaling via the STAT3 pathway. Based on a genomic response element bound by activated STAT3, the decoy molecules bind and inhibit STAT3 dimers. A first-generation STAT3 decoy (S3D) was a linear double-stranded ODN, 15 base pairs in length with free ends. This linear S3D inhibits the growth of solid tumors in preclinical models and is well-tolerated in preclinical models [21,25,26,27,28,29]. In a Phase 0 clinical trial, intratumoral injection of the linear S3D decreased expression of the STAT3 target genes encoding anti-apoptotic Bcl-XL and pro-proliferative cyclin D1 [30]. In an effort to develop a STAT3 decoy formulation more resistant to degradation by nucleases and, thereby, amenable to systemic delivery, hexaethylene glycol spacers were covalently attached to the free ends of linear S3D to create the second-generation cyclic STAT3 decoy (CS3D) [30]. Although CS3D has demonstrated similar biological activity and safety as its linear predecessor, the stability of CS3D in human serum has not been determined [26,30].

In the present study, we investigated the biochemical properties of CS3D. Since complete cyclization of CS3D requires an enzymatic ligation step, we first determined the efficiency of this ligation process. A biotinylated version of the CS3D was also generated, allowing pull-down of intact CS3D from human serum samples and determination of stability. Our results demonstrate that CS3D exhibits a roughly three-fold longer half-life in human serum compared to the first-generation linear S3D, an improvement that will facilitate more effective systemic delivery in humans.

## 2. Results

### 2.1. Efficient Ligation of CS3D

**Figure 1:**
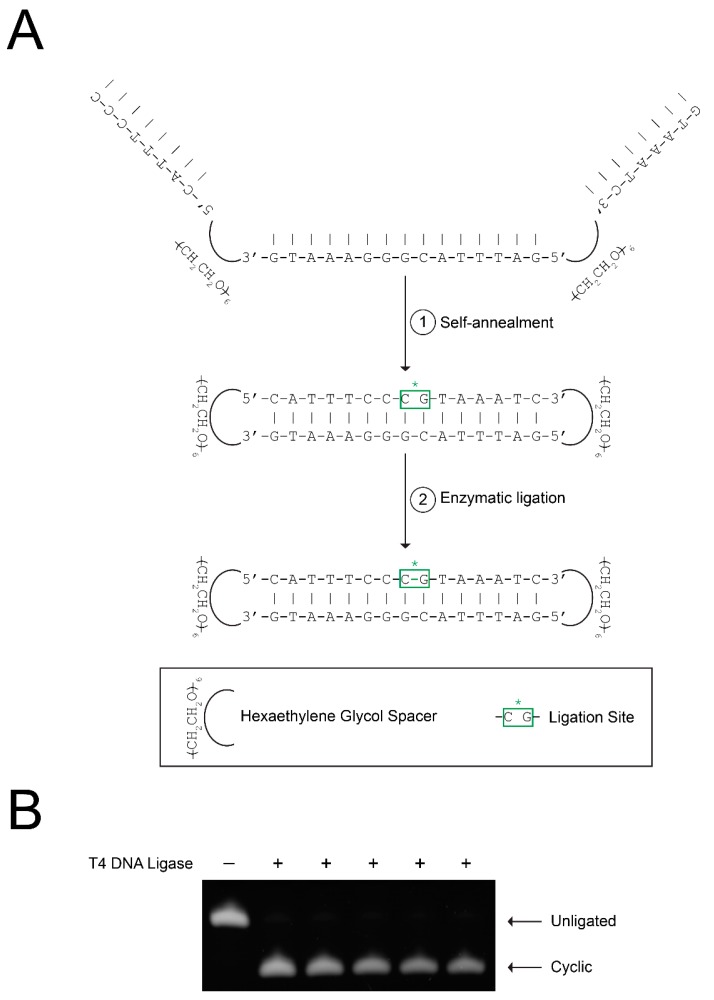
Efficient ligation of cyclic signal transducer and activator of transcription 3 (STAT3) decoy (CS3D). (**A**) Schematic representation of CS3D ligation with T4 DNA ligase. The complementary segments of the single-stranded decoy molecule spontaneously self-anneal. Enzymatic ligation with T4 DNA ligase was used to complete cyclization. (**B**) Incubations were performed in the absence or presence of T4 DNA ligase overnight. Multiple identical ligations (*n* = 5) were simultaneously performed. Samples from each reaction were then electrophoresed on a urea/polyacrylamide gel, stained with SYBR Gold, and quantified by densitometry.

CS3D is initially synthesized as a unimolecular, single-stranded sequence. Following self-annealing at room temperature, enzymatic ligation with T4 DNA ligase is performed to generate the completely cyclic molecule, CS3D (Figure 1A). To investigate the efficiency and consistency of the ligation process, we performed multiple (*n* = 5) small-volume ligations using the same drug stock and identical reaction conditions. Aliquots from each ligation reaction were then subjected to electrophoresis on urea/polyacrylamide gels, followed by staining with SYBR Gold and quantification of band intensity. On average, CS3D was ligated with 94.7 ± 0.5 percent efficiency (Figure 1B). These results suggest that cyclization of CS3D through enzymatic ligation is a consistent and reproducible process.

### 2.2. Biotinylation of CS3D Does Not Affect Ligation Efficiency

**Figure 2:**
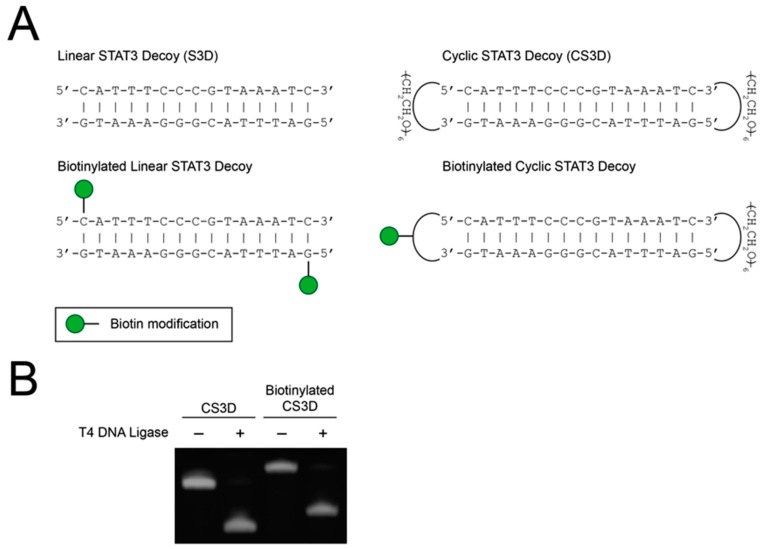
Ligation of cyclic signal transducer and activator of transcription 3 (STAT3) decoy (CS3D) is unaffected by biotinylation. (**A**) Structures of parental and biotinylated STAT3 decoy (S3D) and CS3D. (**B**) CS3D and biotinylated CS3D were incubated with T4 DNA ligase overnight, followed by electrophoresis on a urea/polyacrylamide gel and staining with SYBR Gold.

After establishing minimal variability between CS3D enzymatic ligations, we sought to compare the stabilities of linear and cyclic STAT3 decoys in human serum. Previous studies have demonstrated that covalent modifications to the terminal nucleotides of ODN decoys protect against serum nucleases [31,32,33,34]. We hypothesized that flanking linear S3D with hexaethylene glycol spacers and subsequent cyclization would shield the free ends from nucleolytic degradation in human serum (Figure 2A). To enable quantitative determination of decoy levels in human serum, we developed a pull-down assay to purify the decoys from the serum samples. The assay utilized streptavidin-conjugated agarose resin to efficiently pull down biotinylated versions of S3D or CS3D. In preliminary experiments, we first evaluated whether biotinylation would affect enzymatic ligation of CS3D. As expected, biotinylation increased the molecular weight of CS3D, but had no observable impact on ligation efficiency (Figure 2B).

### 2.3. CS3D Demonstrates Greater Stability in Human Serum than Linear STAT3 Decoy (S3D)

**Figure 3:**
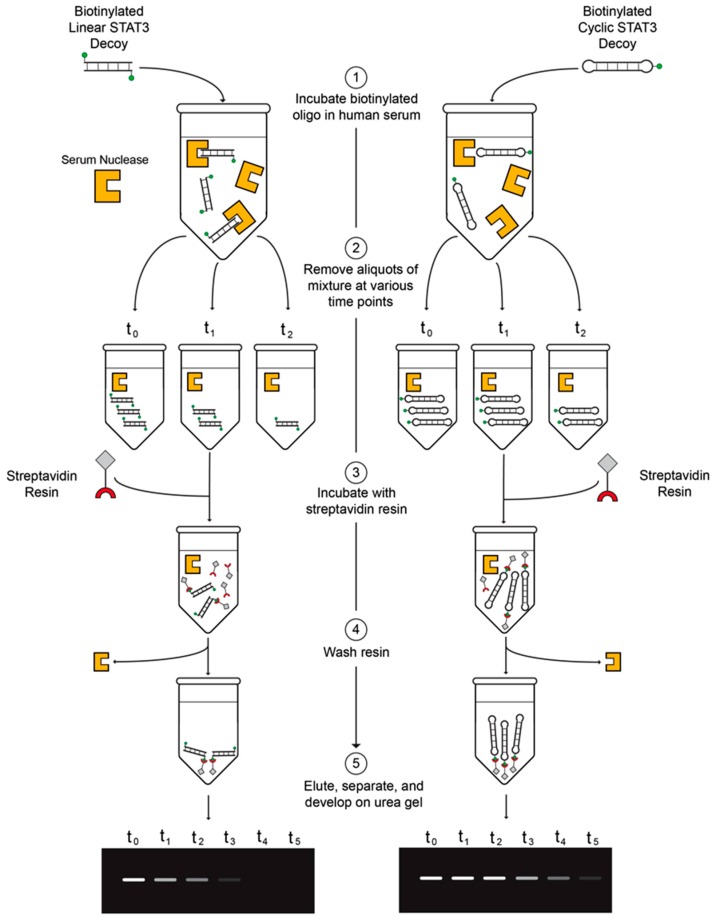
Diagram of the serum stability assay. (1) Incubate biotinylated oligodeoxynucleotide (ODN) in fresh human serum. (2) Remove aliquots of ODN/serum mixture at various time points and store in −80 °C. (3) Incubate streptavidin resin with thawed aliquots of ODN/serum mixture. (4) Pellet and wash three times. (5) Elute by boiling, separate on urea/polyacrylamide gels, and develop with ultraviolet light.

**Figure 4:**
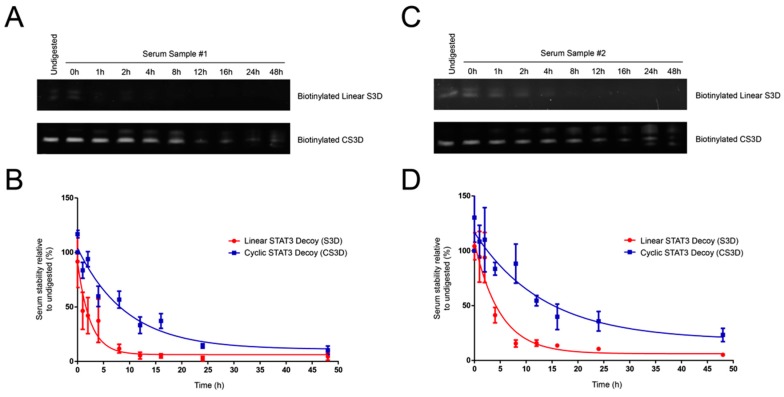
Cyclic signal transducer and activator of transcription 3 (STAT3) decoy (CS3D) exhibits a longer half-life in human serum than linear STAT3 decoy (S3D). (**A**,**C**) Biotinylated S3D and CS3D were incubated in human serum samples #1 or #2 and aliquots were removed at varying time intervals, electrophoresed on urea/polyacrylamide gels, and stained with SYBR Gold. (**B**,**D**) Densitometric analyses were performed on biotinylated S3D and CS3D pulled down from serum. Stability at each time point was expressed as a percent relative to its respective “undigested” control. Undigested control represents decoy suspended in 1X phosphate-buffered saline (PBS) and lacking any serum. Each time point of the S3D and CS3D serum stability assays was performed in triplicate for each sample of human serum.

To determine the stabilities in human serum of the linear first-generation and cyclic second-generation STAT3 decoys, the biotinylated decoys were incubated in fresh human serum for varying lengths of time, pulled down with streptavidin–agarose resin, and then analyzed on urea/polyacrylamide gels (Figure 3). Serum stability assays were performed in two different human serum samples for more reliable assessment of decoy half-lives (Figure 4A–D). The linear S3D exhibited an average half-life of 2.5 ± 1.2 h in the two serum samples (1.7 h in serum sample #1; 3.4 h in serum sample #2). CS3D, on the other hand, exhibited markedly enhanced resistance to degradation with an average half-life of 7.9 ± 2.0 h (6.5 h in serum sample #1; 9.3 h in serum sample #2). These findings underscore the improved stability of CS3D and its potential for clinical application as a systemically delivered agent. 

## 3. Discussion

Numerous solid tumor malignancies have been shown to exhibit oncogenic dependency on STAT3 [35,36]. However, no direct STAT3 inhibitors are currently used in clinical practice. Cyclic STAT3 decoy (CS3D), a double-stranded oligodeoxynucleotide (ODN) that competitively inhibits STAT3 binding to genomic response elements, has been shown to exert selective cytotoxicity against cancers with hyperactivated STAT3 [26,30,37]. To date, the stability of CS3D in human serum has remained unknown. In this study, we utilized a novel pull-down approach to measure the serum stabilities of CS3D, as well as an earlier linear version of the STAT3 decoy. By comparing the respective half-lives of the cyclic and linear STAT3 decoys, we determined that cyclization resulted in substantially improved stability in human serum. We further determined that the process of enzymatic ligation used to generate the cyclized CS3D is highly efficient and can likely be scaled up to generate large quantities of the inhibitor. Translating CS3D to the clinic will augment current treatment strategies for cancers that are characterized by dependence on STAT3 signaling. Moreover, successful application of CS3D will provide a foundation for the development and use of ODN decoys to target transcription factors in disease states including and beyond cancer.

Transcription factor decoys comprise a class of nucleic acid-based agents that modulate the cellular activity of transcription factors, proteins that are often considered undruggable [38]. Decoys are short double-stranded ODNs designed to mimic the genomic sequences in target gene promoters that are recognized by transcription factors [39]. Although decoys have been studied in clinical trials for venous bypass graft vasculopathy and head and neck squamous cell carcinoma (HNSCC), instability represents a major translational hurdle because extracellular and intracellular nucleases rapidly degrade the free ends of linear ODN decoys [30,40,41,42]. In an effort to minimize such degradation, modifications at the free ends of decoy ODNs have been used to improve resistance against enzymatic activity. These modifications include the use of phosphorothioate linkers, unnatural nucleotides, and polyethylene glycol attachments [31,32,33,34]. Prior studies to assess the stabilities of modified ODNs have primarily focused on laboratory-generated aqueous solutions containing defined levels of human nucleases, whereas the current study assessed decoy stability in human serum samples. Moreover, we developed and utilized a novel pull-down assay, allowing us to easily purify intact, biotinylated decoys from the serum. Our findings of improved stability with the cyclic STAT3 decoy confirm the value of modifying or removing the free ends of decoy ODNs.

Although our results suggest that CS3D may be amenable to intravenous delivery in humans, the optimal approach for targeted delivery to diseased tissue remains unclear. Strategies that have been developed to enhance delivery include topical ointments, and the use of glycopolymers and nanoparticles, among others [43,44,45]. Another method that has proven successful involves the use of an ultrasound-targeted microbubble destruction (UTMD) system [46]. In this system, ODNs are packaged into microbubbles that are delivered intravenously. An external ultrasound probe at the desired delivery site facilitates rupture of the microbubbles, resulting in localized delivery. A previous study using UTMD-mediated delivery of CS3D has demonstrated intratumoral uptake in a murine model [47].

In summary, the use of cyclic ODN decoys may provide an effective strategy for targeting transcription factors, including STAT3. However, no transcription factor decoys have yet been translated to clinical practice. Our results reveal that fully cyclized CS3D can be efficiently generated via enzymatic ligation. Moreover, CS3D demonstrates improved stability in human serum relative to linear decoy with free ends. These findings will facilitate further clinical development of CS3D and provide evidence supporting the potential use of transcription factor decoys as viable therapeutic agents.

## 4. Materials and Methods

### 4.1. Synthesis of Biotinylated Linear STAT3 Decoy (S3D) and Biotinylated Cyclic STAT3 Decoy (CS3D)

Biotinylated linear S3D was synthesized as two complementary single-stranded oligodeoxynucleotides (ODNs) by Sigma-Aldrich (St. Louis, MO, USA). Biotinylated CS3D was synthesized as a unimolecular single-stranded ODN by Integrated DNA Technologies (Skokie, IL, USA). S3D and CS3D were reconstituted in normal saline as 800 μM stock solutions and stored at −20 °C.

### 4.2. Ligation of Cyclic STAT3 Decoy (CS3D) and Biotinylated CS3D

15 μL of single-stranded oligodeoxynucleotides (ODNs) were incubated with 3 μL of T4 DNA ligase (400,000 U/mL; New England BioLabs, Ipswich, MA, USA, M0202S) and 2 μL of 10X T4 DNA ligase reaction buffer (New England BioLabs, B0202S) overnight at room temperature. Ligated material was subsequently diluted to 1 μM, incubated with DNA loading buffer at 60 °C for 5 min, and electrophoresed on 15% Tris-Borate-EDTA (TBE) urea/polyacrylamide gels. The ODNs were detected by incubating gels in 1X SYBR Gold solution (Thermo Fisher Scientific, Waltham, MA, USA, S11494) for 1 h at 4 °C in the dark, followed by exposure to ultraviolet light and image detection using Azure c600 (Biosystems, Dublin, CA, USA). The relative densities of ligated and unligated ODN bands were quantified using Image Studio Lite v5.2.5 (LI-COR Biosciences, Lincoln, NE, USA).

### 4.3. Annealing of Biotinylated Linear STAT3 Decoy (S3D)

Complementary sense and antisense strands of biotinylated linear S3D were incubated in equimolar concentrations at 100 °C for 1 h, followed by annealing at room temperature overnight. The annealed oligodeoxynucleotides (ODNs) were stored at 4 °C.

### 4.4. Serum Stability Assay

The stability of biotinylated ODNs in human serum was determined following incubation and pull-down of the intact ODN [30,48]. Briefly, human serum was obtained from two individuals in compliance with the Institutional Review Board of the University of California, San Francisco (IRB #14-15342). Biotinylated ODNs were incubated in 100% human serum at a final concentration of 0.05 μg/mL. After varying lengths of time, aliquots (20 μL) were removed and immediately stored at −80 °C. After the final time point, each aliquot was incubated with streptavidin-coated agarose resin (Pierce Streptavidin Resin, Thermo Fisher Scientific, 20347) for 60 min at room temperature, pelleted by centrifugation, and washed 3 times in 3X phosphate-buffered saline (PBS) containing 0.05% Tween-20. The pelleted resin was then resuspended in DNA loading buffer, followed by heating at 95 °C for 4 min to elute the ODNs. Supernatants were electrophoresed on 15% TBE urea/polyacrylamide gels and visualized as described above. The optical densities of the samples were compared to the optical densities of corresponding biotinylated ODNs suspended in 1X PBS that were not incubated in serum.

### 4.5. Statistical Analysis

Analyses of the serum stabilities of S3D and CS3D were performed using GraphPad Prism 5.04 (La Jolla, CA, USA).

## Abbreviations

S3D	linear STAT3 decoy
CS3D	cyclic STAT3 decoy
ODN	oligodeoxynucleotide
STAT3	signal transducer and activator of transcription 3
JAK	Janus kinase
HNSCC	head and neck squamous cell carcinoma
UTMD	ultrasound-targeted microbubble destruction
PBS	phosphate-buffered saline
